# COVID-19 Impact on Diagnostic Innovations: Emerging Trends and Implications

**DOI:** 10.3390/diagnostics11020182

**Published:** 2021-01-27

**Authors:** Anne O. Oyewole, Lucy Barrass, Emily G. Robertson, James Woltmann, Hannah O’Keefe, Harsimran Sarpal, Kim Dangova, Catherine Richmond, Dawn Craig

**Affiliations:** 1 National Institute for Health Research (NIHR) Innovation Observatory, Newcastle University, Newcastle NE4 5TG, UK; lucy.barrass@io.nihr.ac.uk (L.B.); emily.robertson@io.nihr.ac.uk (E.G.R.); James.Woltmann@io.nihr.ac.uk (J.W.); nho11@newcastle.ac.uk (H.O.); Harsimran.Sarpal@io.nihr.ac.uk (H.S.); kim.dangova@io.nihr.ac.uk (K.D.); catherine.richmond@ncl.ac.uk (C.R.); dawn.craig@io.nihr.ac.uk (D.C.); 2 Evidence Synthesis Group, Population Health Sciences Institute, Newcastle University, Newcastle NE2 4AX, UK

**Keywords:** COVID-19, SARS-CoV-2, diagnostic tests, IVDs, immunoassays, rapid diagnostic tests, molecular assays, horizon scanning

## Abstract

Diagnostic testing remains the backbone of the coronavirus disease 2019 (COVID-19) response, supporting containment efforts to mitigate the outbreak. The severity of this crisis and increasing capacity issues associated with polymerase chain reaction (PCR)-based testing, accelerated the development of diagnostic solutions to meet demands for mass testing. The National Institute for Health Research (NIHR) Innovation Observatory is the national horizon scanning organization in England. Since March, the Innovation Observatory has applied advanced horizon scanning methodologies and tools to compile a diagnostic landscape, based upon data captured for molecular (MDx) and immunological (IDx) based diagnostics (commercialized/in development), for the diagnosis of SARS-CoV-2. In total we identified and tracked 1608 diagnostics, produced by 1045 developers across 54 countries. Our dataset shows the speed and scale in which diagnostics were produced and provides insights into key periods of development and shifts in trends between MDx and IDx solutions as the pandemic progressed. Stakeholders worldwide required timely and detailed intelligence to respond to major challenges, including testing capacity and regulatory issues. Our intelligence assisted UK stakeholders with assessing priorities and mitigation options throughout the pandemic. Here we present the global evolution of diagnostic innovations devised to meet changing needs, their regulation and trends across geographical regions, providing invaluable insights into the complexity of the COVID-19 phenomena.

## 1. Introduction

**Figure 1:**
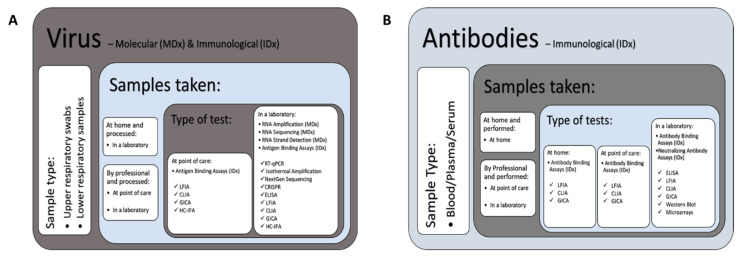
Classification and sample type for molecular (MDx) and immunological (IDx) diagnostics developed to target the virus (antigen), or antibodies (IgG/IgM) produced in response to the virus. (**A**) MDx and virus IDx and (**B**) Antibody IDx. (**A**) MDx detect the antigen (viral nucleic acids) in swab samples taken from an individual’s upper or lower respiratory tract. Swabs may be taken at home by the individual or by a professional and sent for testing in a laboratory. Virus IDx use antibodies to detect proteins found on the surface of the virus and are typically performed using point-of-care (POC) tests. (**B**) Antibody IDx detect antibodies to the virus in blood samples. A subset of these tests can be performed at home by an individual, whilst others are performed by healthcare professionals (POC or sample sent to laboratory testing facilities). Abbreviations: LFIA = lateral flow immunoassay, CLIA = chemiluminescence immunoassay, GICA = colloidal gold immunochromatography, HC-IFA = hybrid-capture immunofluorescence analysis, RNA = ribonucleic acid, RT-qPCR = quantitative reverse transcriptase polymerase chain reaction, CRISPR = clustered regularly interspaced short palindromic repeats, ELISA = enzyme-linked immunosorbent assay.

The current coronavirus disease 2019 (COVID-19) pandemic caused by severe acute respiratory syndrome coronavirus 2 (SARS-CoV-2) presents an unprecedented global challenge [1,2] that has reshaped activities across the diagnostic innovation landscape [3]. As SARS-CoV-2 is a respiratory virus, in vitro diagnostic (IVDs) medical devices sit at the heart of SARS-CoV-2 diagnosis and decisions concerning clinical management [3,4,5,6,7]. According to Market Data Forecast the global IVD market is valued at $70 billion (USD) and includes diagnostic technologies, platforms and reagents to detect and diagnose disease causing pathogens [8]. There are several classes of IVDs, including molecular and immunological tests, which is where much of the focus of diagnostic innovations have been concentrated during the pandemic [6]. Molecular diagnostics (MDx) developed for SARS-CoV-2 (Figure 1A) require swabs from the individual’s respiratory system [9,10]. Swabs may be taken by the individual at home, but more frequently are taken by professionals, helping to ensure that a thorough sampling technique is used [10]. This increases the likelihood that samples will contain a high enough titre for detection. The sample can then be tested with MDx to detect viral genomic material in a laboratory or point-of-care (POC) test [11]. Quantitative reverse transcription polymerase chain reaction (RT-qPCR) is the most widely used molecular technique for the diagnosis of SARS-CoV-2 in hospitals, laboratories and other health care facilities [2,9,12,13], and requires sophisticated equipment and trained laboratory staff [2]. The severity of the crisis and increasing capacity and logistical issues associated with this method (including shortages in PCR reagents [14,15,16,17,18] and the time taken to process samples [14,15]), led to an increase in the development and adoption of alternative diagnostic solutions (e.g., molecular [14,19] and immunological-based solutions [20,21]) to supplement PCR-based detection [16,22,23,24,25]. 

Growing demand for rapid and easy-to-use diagnostics to facilitate testing outside of laboratory settings [6,19,26,27,28], also resulted in a drive in the development of immunological diagnostics (IDx) that detect the presence of viral surface antigens utilizing an antibody (virus IDx), as these tests produce faster results than MDx (Figure 1A) [29,30,31]. Immunological tests have also been designed to detect the presence of antibodies (IgG/IgM) to a pathogen (antibody IDx) in the serum and plasma drawn from a blood sample (Figure 1B) [11,20,21,32,33]. Antibody testing can be performed if it is believed that the individual has previously had the virus. Antibodies are produced by the body’s immune system in response to infection and circulate in the blood stream for some time after the infection has been cleared [34]. For some infections, antibodies can provide lifelong immunity, however, studies have shown that the immune response to SARS-CoV-2 could last between 5–7.5 months [35,36]. As antibody tests only require a small blood sample, often a simple finger prick [20,33], these tests are often developed in a format which can be conducted at home, similar to a pregnancy test. Despite this, the majority of antibody tests are approved for healthcare professionals use only. Whilst MDx and virus IDx help determine who is currently infected, antibody IDx provide a broader picture of the extent of the pandemic (i.e., prevalence of the disease) and the potential level of immunity within the population. The ability of various organizational entities to accelerate the development of diagnostic tests for SARS-CoV-2 in a short period of time, is a result of the coronavirus genome sequence being published within a few weeks of the virus being initially detected [37,38]. In contrast, it has typically taken months to identify and develop tests for other pathogens responsible for outbreaks, for example the 2002–2003 SARS [39].

**Figure 2:**
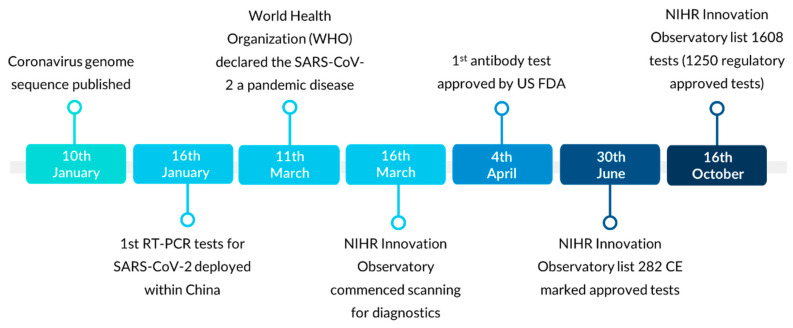
Severe acute respiratory syndrome coronavirus 2 (SARS-CoV-2) diagnostic timeline. Timeline of the main developments and outputs since the beginning of the coronavirus disease 2019 (COVID-19) pandemic. The European Conformité Européene (CE) mark and the United States Food and Drug Administration (US FDA) approval mark, indicate that in vitro diagnostic (IVD) medical devices meet the regulatory requirements for the jurisdiction.

As countries worldwide entered the first wave of the pandemic, aggressive diagnostic testing strategies became critical, especially in the absence of an effective therapy or vaccine for SARS-CoV-2 [6,40]. In response, the National Institute for Health Research (NIHR) Innovation Observatory commenced horizon scanning for emergent diagnostic tests that were commercialized or in development for the diagnosis of SARS-CoV-2 (antigen or antibody) (Figure 2). In addition to our efforts, we also identified other organizations such as the Foundation for Innovative New Diagnostics (FIND) [41] that listed SARS-CoV-2 diagnostics. At a national level our horizon scanning produced diagnostic innovation pipeline reports highlighting key insights into the evolution of technological innovations, their regulation, as well as emerging trends across geographical regions. Our comprehensive intelligence served as a timely source assisting UK agencies, policymakers and commissioners in assessing priorities and mitigation options in response to testing challenges. For example, our intelligence was valuable in anticipating and identifying tests with the potential to alleviate pressures on PCR-based resources and supply chains. We also supported the identification of ‘direct-to-consumer’ testing services for SARS-CoV-2 as part of a regulatory review, as the products and services remain controversial and pose ethical concerns on the grounds of the harm they may potentially cause to consumers [42,43,44,45,46]. This paper outlines key technological and regulatory trends for COVID-19 diagnostics and highlights key implications and challenges in this shifting health technology landscape.

## 2. Materials and Methods 

The Innovation Observatory is a national horizon scanning facility funded by England’s NIHR, and in March 2020, began an active horizon scan for IVDs that could be used in the diagnosis of SARS-CoV-2. The objective of this horizon scan was to alert regulators, policymakers and commissioners in the UK of approved and emerging diagnostic technologies from across the world; as well as in-house laboratory-developed diagnostics, kits; and related test methods for SARS-CoV-2, to support containment efforts to mitigate the COVID-19 outbreak impact on NHS services. The intelligence gathered was also published on our dedicated COVID-19 Diagnostic Dashboard to allow for open access, ensuring that other interested stakeholders, including the public could access our intelligence on this growing topic of interest [47]. 

The horizon scan methodology developed by the Innovation Observatory involved the identification of information sources that detected ‘signals’ for diagnostic technologies for SARS-CoV-2. The collection of sources that were identified were systematically scanned and monitored for intelligence using a combination of traditional scanning methods (manual), automated and novel text-mining techniques. Search strategies were created for specific sources (e.g., clinical trial registries) and combined key terms related to SARS-CoV-2. Based on successive screening of sources (i.e., identification of diagnostics), intelligence was extracted and imported for further data processing. The data presented in this publication was collected between 16 March 2020 and 16 October 2020 (inclusive). This period of diagnostic development has been categorized into 3 key phases: The initial Rapid Growth Phase (March–April), followed by the Deceleration Phase (May–July) and Renewal/Deceleration Phase (August–October).

### 2.1. Identification 

Diagnostic technologies for use in the diagnosis of SARS-CoV-2 were identified by systematically scanning primary and secondary publicly available sources. These sources (full list available on our website [47]) of information spanned national and international clinical trial registries (e.g., U.S. National Institutes of Health (NIH) clinical trials registry), publications by national regulatory bodies and non-governmental organizations (e.g., FIND), available commercial reports and a range of online news and MedTech websites. The inclusion criteria for diagnostics technologies reported in this work may introduce bias, due to the differences in the regulatory requirements used to assess diagnostic technologies (including in-house laboratory-developed diagnostics and kits) across jurisdictions. In addition to the identification of diagnostic technologies, a separate horizon scan was conducted for ‘Direct-to-consumer Service Tests’ (antigen or antibody-based) available in the UK. The guidance on the regulations of these service tests (including clinical accuracy) and their availability differed based on the jurisdiction in which these tests were being marketed.

### 2.2. Inclusion and Date Extraction 

As diagnostic tests may be sold or imported by multiple companies across different countries, it was important to determine the originating developer/manufacturer to avoid the inclusion of duplicate records for the same technology and to link importers to the primary company (i.e., manufacturer). Once verified as a new record, the diagnostic test was added, and further investigation was undertaken to retrieve supporting information. The information collected for each technology was categorized, and included, inter alia: company; product name; diagnostic category; type of assay; sample type; detection target; assay method; development status and regulatory status. Any information pertaining to regulatory approval of a technology obtained from company websites, press releases, and/or sales pages was verified against regulatory authority sources. 

### 2.3. Monitor

Due to the rapidly evolving COVID-19 diagnostic landscape, the information for each technology was closely monitored and updated as new intelligence emerged from daily scans. Daily review of specific national regulatory bodies [41,48,49,50,51,52,53,54,55,56,57] and outputs from the World Health Organization (WHO) [58] were performed to ensure that the regulatory information tracked was up-to-date and regularly re-verified.

## 3. Results

### 3.1. Severe Acute Respiratory Syndrome Coronavirus 2 (SARS-CoV-2) Diagnostic Technology Landscape 

**Figure 3:**
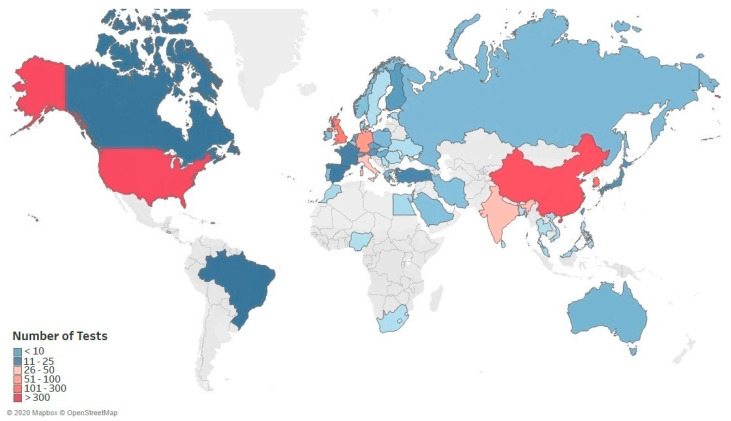
Global SARS-CoV-2 diagnostics landscape. The map provides an overview of the global scale of diagnostic technologies that have been developed for SARS-CoV-2, based on the Innovation Observatory’s comprehensive dataset of 1608 diagnostics, either approved or in the development phase for clinical use. The United States (408) and China (391) held dominant positions in the diagnostic market, accounting for 49.7% of diagnostics developed.

**Figure 4:**
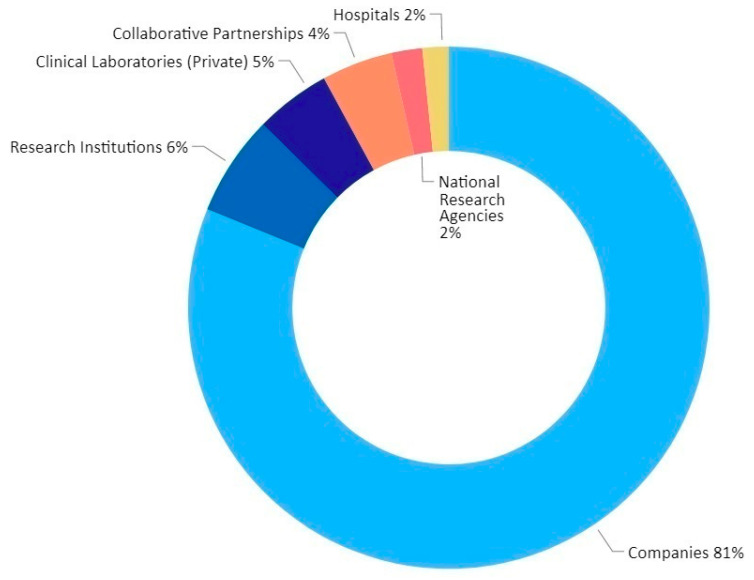
Developers of diagnostic technologies for SARS-CoV-2 (global perspective). Distribution of development of diagnostic technologies (e.g., IVDs) according to the entity type.

The growing prevalence of infectious diseases is a major factor driving the growth of the global IVDs market for infectious disease. The COVID-19 outbreak like with other highly contagious infections (e.g., SARS, Ebola virus disease), has led to the rapid development and adoption of diagnostics, as part of the critical response plan. Thus, the global implementation of testing strategies has been a key driver for the changing diagnostics landscape (Figure 3). The Innovation Observatory’s strategic horizon scanning allowed the establishment of a comprehensive global dataset of 1608 IVDs that were produced by 1045 developers in 54 countries [47]. Developers were from a range of different sectors but overall companies (SMEs and large enterprises) dominated the diagnostic innovation landscape, producing 81% of diagnostic technologies (e.g., IVDs) (Figure 4). The companies in the dataset are primarily based in Europe, North America and Asia. Other developers included, research institutions (6%), private clinical laboratories (5%), collaborative alliances (4%) and national research agencies (2%) who made up 17% of diagnostics technologies developed for COVID-19, whilst the development of diagnostics in hospitals made up just 2%. In the UK, the proportion of companies contributing to the development of SARS-CoV-2 tests was 63% and there was evidence of a concentration of collaborative alliances (19%) and research institutions (13%), which we anticipate strengthened the capacity to respond to the growing demand for diagnostics locally. In contrast, the proportion of companies contributing to the development of SARS-CoV-2 tests in the United States was 66% and in China 94%. 

### 3.2. Global Trends in SARS-CoV-2 Diagnostic Market 

**Figure 5:**
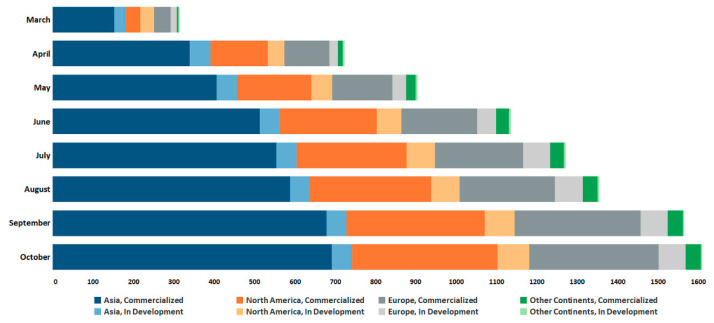
Global comparison of trends in SARS-CoV-2 diagnostic test development (March–October 2020). Diagnostic technologies (cumulative data) for SARS-CoV-2 (commercialized or in development phase) between March and October, produced across geographical regions (Asia, North America, Europe and Other Continents inclusive of South America, Africa and Oceania). Overall, the data demonstrate that Asia had the strongest continental response to the development of diagnostics, accounting for 46.1% of the global diagnostic landscape.

The global trends presented in Figure 5 highlight the fast-changing and dynamic global response to the development of diagnostic solutions in response to COVID-19 during March to October 2020. Our analysis of this landscape revealed a precipitous surge in the development of diagnostic solutions during March and April (Rapid Growth Phase), correlating with the high demand for IVDs due to the prevalence of the respiratory disease [4,6,25,28]. The rate of diagnostic technologies emerging for SARS-CoV-2 during this phase averaged 120 per week across all regions. A closer look at the data revealed that overall, the highest proportion of diagnostics were developed in Asia (46.1%), followed by North America (27.3%) and the European Union (EU, 24.1%). The marked increase in diagnostic activity in countries in the region of Asia is linked to these countries being affected early in the pandemic [59,60], and their implementation of aggressive large-scale testing strategies [7,59,61], which demanded a greater numbers of technologies to be developed by entities such as companies in this region [59,62,63,64,65,66]. Interestingly, there appeared to be on average a lower proportion of diagnostic solutions in the ‘development stage’ compared to those in the ‘commercialization stage’ in Asia (9.7%) in contrast to North America (23.6%) throughout the pandemic. Evidence appears to indicate that this is the result of a higher level of mature technologies being employed for molecular-based tests in Asia, thus less time was required for the development of these technologies [64,67,68,69,70]. 

**Figure 6:**
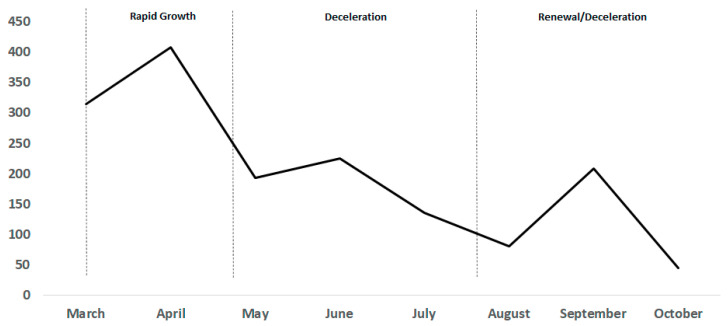
Trends in SARS-CoV-2 diagnostics development divided into 3 phases: 1) Rapid Growth Phase; 2) Deceleration Phase and; 3) Renewal/Deceleration Phase. The development activity of diagnostics for SARS-CoV-2 was monitored between the 16th March 2020 and 16th October 2020 (inclusive). This period of diagnostic development has been categorized into 3 key phases: the initial Rapid Growth Phase (March–April) is characterized by the increase in the development of diagnostic technologies. The phase was driven by various factors (e.g., research and development (R&D) capacity, demand and supply), and eventually slowed down and reached its peak. This phase was followed by the Deceleration Phase (May–July) which was marked by the gradual decrease in the number of diagnostics emerging, before a spike (September) in the ‘Renewal/Deceleration Phase’ (August–October).

Whilst Asia dominated the diagnostic landscape, the role of companies in Europe and North America (namely the United States) became more evident as the pandemic reached these regions and the need to increase testing capacity became more urgent [17,25,71,72,73,74,75]. As the pandemic progressed [76], the rate of diagnostic development activity gradually slowed down between May and July (Decline Deceleration Phase) (Figure 6). During this phase the rate of diagnostic technologies emerging averaged 46 per week across all regions, around a third of the number of tests developed in the Rapid Growth Phase (Figure 6). This deceleration in development likely reflects the saturation of the COVID-19 diagnostic market with commercialized solutions offered by the majority of companies/developers in the industry, and the existing deployment of a high level of diagnostics into national services and operations [77]. Interestingly, our data revealed a resurgence in both MDx and IDx development activity in September, with the number of tests identified similar to that of June (Figure 6). Development activity in IDx appeared to be the main contributor to this growth, accounting for 64% of diagnostics identified in September. The striking surge, however, was immediately followed by a considerable deceleration in diagnostic activity (Renewal/Deceleration Phase). 

### 3.3. Global Trends in SARS-CoV-2 MDx and IDx Solutions 

**Figure 7:**
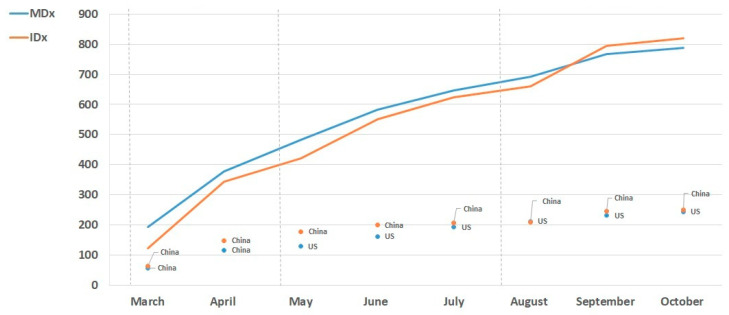
Global comparison of trends in SARS-CoV-2 Molecular (MDx) and immunological (IDx) diagnostics development (March–October 2020). Worldwide trends in MDx and IDx development activity for SARS-CoV-2 (cumulative data). MDx and IDx development increased in a stepwise fashion between March and October 2020. Overall, a slightly higher level of MDx solutions emerged compared to IDx solutions, however a shift in development activity was observed (August –September) with a higher number of IDx solutions in the diagnostic landscape. The United States and China produced the highest level of MDx and IDx technologies (respectively).

Our analysis of the global MDx and IDx landscape revealed that in the Rapid Growth Phase, on average molecular-based diagnostics (i.e., MDx) accounted for 52.4% of diagnostic solutions identified. The focus on MDx solutions in the first wave of the pandemic is linked to the prevalence of the disease and the urgency of early detection and rapid response. Shortages of PCR consumables [14,15,16,17,18] coupled with laboratory testing capacity issues (i.e., sample processing time) [4,6,25,28] caused a surge in the technological advancements of a variety of molecular-based techniques including clustered regularly interspaced short palindromic repeats (CRISPR), chemiluminescent assays (CLIA) and lateral flow immunoassay (LFIA) [5,6,14,15,16,17,18,27,68]. Furthermore, the availability and distribution of these tests was accelerated by regulatory agencies issuing interim approval mechanisms to address this large-scale public health emergency [48,70]. Figure 7 shows the diagnostic activity trends of MDx and IDx solutions for SARS-CoV-2. Overall, the trends provide interesting insights into changing diagnostic activity of developers, which may have been driven by the demands of national stakeholders [22,24,25]. As the pandemic progressed, we observed a shift in the development activity of MDx and IDx. A higher proportion of IDx emerged during the Renewal/Deceleration Phase (August–October), accounting for on average 62.7% of diagnostic development (Figure 7). This increase in the development activity of immune-based detection solutions appears to correlate with the severity of the disease, and the inability of molecular-based diagnostics (e.g., RT-qPCR) to detect past infection [78,79,80,81,82]. As some countries emerged from the first wave of the pandemic, whilst others entered the second wave of the pandemic, priorities shifted to active surveillance strategies, to understand the geographical spread and severity of the disease in the population [79,80,81,82]. Overall, Asia developed the highest proportion of MDx (42.6%) and IDx (49.5%) diagnostic technologies, almost twice the amount of MDx (22.9%) and IDx (25.2%) technologies than Europe. In North America, the development of MDx and IDx accounted for 33% and 21.9% (respectively), whilst the rest of the world represented 1.5% (MDx) and 3.4% (IDx). Interestingly, on a national level the United States produced the highest level of MDx technologies (253) and the second highest number of IDx technologies (169). The United States IVD market is valued at $9 million (USD) which is significantly greater than China’s market value of $4 million (USD) [83,84]. The high proportion of COVID-19 MDx and IDx technologies that emerged from the United States can be attributed to their greater market share and the large number of diagnostic companies (including large enterprises) based in this region, giving rise to a higher potential for the development of solutions to detect the virus [83,84]. Other countries with a strong focus on MDx and IDx solutions included China (142; 249) and South Korea (94; 85) followed by the UK, Germany and Singapore [47]. 

When comparing the distribution of MDx and IDx by region, countries in South America, Africa and Oceania were observed to have developed the fewest technologies. 

### 3.4. Global Trends in SARS-CoV-2 Rapid Diagnostic Solutions 

**Figure 8:**
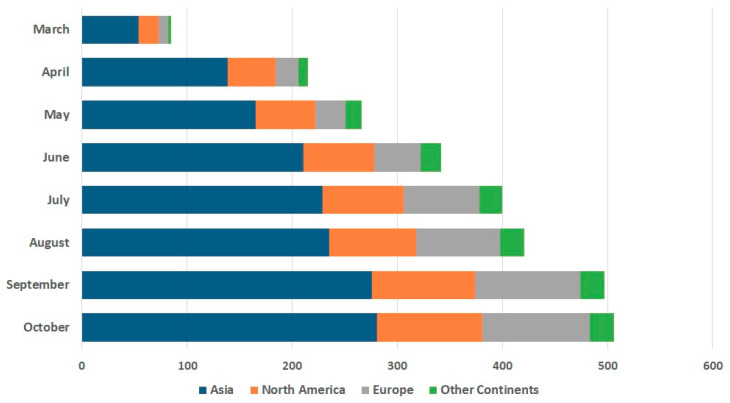
Global comparison of trends in SARS-CoV-2 rapid diagnostics (MDx and IDx) development (March–October 2020). The development of rapid diagnostics for SARS-CoV-2 precipitously increased around the world. A total of 506 rapid diagnostic solutions were identified between March and October 2020. Asia showed the highest development activity for rapid diagnostic tests accounting for 55.5% of all rapid solutions. IDx (i.e., immunological-based detection methods) accounted for 95.3% of rapid diagnostics produced compared to MDx (4.7%).

As countries struggled to contain the spread of COVID-19, the rapidity and ease of use of diagnostic tests became important criteria [16,22,23,24,25,31,85,86]. Healthcare systems across the world accelerated the development and or adoption of rapid diagnostics to expand testing capacity (i.e., supplement PCR-based detection) [16,22,23,24,25,86]. Global partnerships also enabled low and middle-income countries who have limited testing infrastructure (e.g., PCR-based detection and trained staff) to access affordable rapid tests [22,23]. Rapid diagnostics are defined as tests which are relatively simple to perform and interpret, requiring limited training and providing fast results [33,87,88]. Many diagnostic solutions were developed to detect the ‘antigen’ (including genomic material (MDx) and viral proteins (virus IDx) [31,85,89,90,91,92,93] or antibodies (IgG/M) produced against the virus (antibody IDx) [33,94,95]. The data in Figure 8 shows a marked increase in the development of rapid diagnostic solutions worldwide in the Rapid Growth Phase. This increase corresponds with the greater operational need for large scale testing [4,7,31,92,96,97,98], specifically, the need to deploy both rapid ‘antigen’ and ‘antibody’ diagnostic tests in community settings to reduce waiting time for results and to relieve the burden on healthcare services [90,99,100]. A closer look at the data presented in Figure 8 reveals that Asia produced the highest proportion of rapid diagnostic solutions (55.5%) followed by Europe (20.2%) and North America (19.7%). On a national level China (180), followed by the United States (86), South Korea (49) and UK (36) were the most prominent players in the development of rapid diagnostics for SARS-CoV-2. 

In total, 506 rapid diagnostic solutions were identified, and these comprised of 482 IDx (antigen or antibody-based) and 24 MDx (antigen-based) solutions. Typically, rapid tests are performed in under 30 min [31] and we observed that the pipeline of technological innovation produced solutions with considerably shortened result times. A total of 283 rapid diagnostics were reported to provide results in 6–15 min, whilst 24 tests claimed to provide results in 5 min or less, with 7 of these tests reporting to deliver results in under 1 min. The majority of IDx technologies produced focused on antibody (IgG/M) detection (82.78%). A major challenge associated with many rapid immunological-based diagnostics (antibody and antigen) is the variability in their clinical performance. Unlike RT-qPCR where the genetic material of the virus is amplified, immunological methods do not amplify their protein signal and are, therefore, subject to weaker signals and thus lower sensitivity levels [99]. Rapid diagnostics that detect viral proteins in patient samples (e.g., saliva/nasal swab) are also associated with other challenges, including the identification of effective antibodies that bind to a single viral protein and antibody cross-reactivity [85,101]. 

### 3.5. Evolving SARS-CoV-2 Diagnostics Regulatory Landscape 

**Figure 9:**
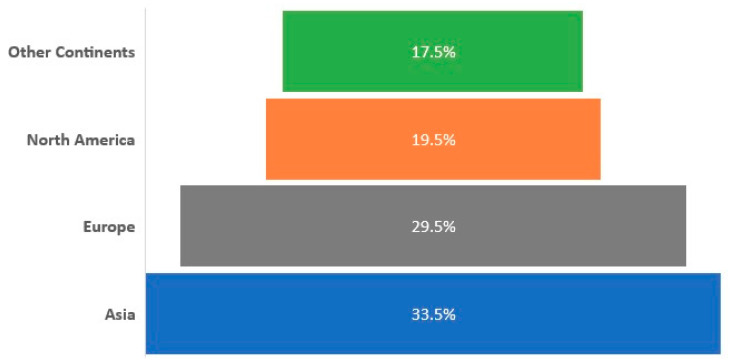
Comparison of SARS-CoV-2 regulatory landscape. An overview of the global scale and variation in regulatory approval of diagnostic technologies for SARS-CoV-2. Regulatory agencies worldwide adapted their guidelines to ensure the accelerated assessment of diagnostics. Overall, a total of 1050 diagnostic technologies received regulatory approvals up to and including 16 October 2020, with Asia granting the highest number of approvals (33.5%), followed by Europe (29.5%), North America (19.5%) and Other Continents (South America, Africa and Oceania) (17.5%).

The rapid development and deployment of diagnostic technologies presented a challenge for existing regulations for IVDs [25,86]. Subsequently, regulatory agencies across the world adapted their guidelines and policies to facilitate the accelerated evaluation of MDx and IDx and expedite their market access [42,48,49,55,102]. On the 4th February 2020 the United States Department of Health and Human Services issued an emergency use authorization (EUA) specific to the development of IVDs for SARS-CoV-2. Due to these revisions, more than 280 SARS-CoV-2 diagnostic technologies were approved by the United States FDA by the end of October 2020 [48]. Figure 9 provides an overview of the geographical variations in regulatory approvals for diagnostic solutions amid COVID-19. Up until 16 October, regulatory agencies from across the world had issued 1697 approvals for 1050 diagnostic technologies. Our data indicated that 70.8% of diagnostic technologies had received approval in one jurisdiction, and in contrast only 29.2% had obtained regulatory approvals in two or more jurisdictions. Regulatory approvals in Asia accounted for nearly 34% of the total number of approvals granted. Experts have suggested that the high proportion of approvals in this region may be due to the rigorous COVID-19 testing strategies implemented to contain the spread of the infection [7,59,64,68,75,103,104]. The number of CE (Conformité Européene) marked diagnostic technologies in Europe accounted for 29.5%. North America ranked third in regulatory approvals granted for diagnostic technologies (19.5%), followed by South America (10.2%) and Oceania (5.8%). Regulatory approval in Africa accounted for 1.5% of testing solutions which may be due to the limited regulatory agency resources to efficiently and effectively assess diagnostic technologies [105,106,107], in addition to limited testing infrastructure (e.g., technology, trained staff and facilities) [105,106,107,108,109]. 

### 3.6. Direct-to-Consumer SARS-CoV-2 Service Tests

The number of companies providing direct-to-consumer tests is growing around the world [43,110,111]. Tests for home blood collection and lab based-immunological tests (i.e., antibody tests), home-based saliva sampling for infection and genomic testing are marketed to consumers online or in stores [43,112,113,114,115]. We collected data on 73 providers offering 99 tests in the UK up until 16 October [116,117,118]. Test targeting virus detection (e.g., swab/saliva tests) accounted for 58.6%, whilst antibody tests accounted for 41.4% of tests. Consumer service tests such as genetic testing have grown in popularity over the last decade, with companies offering predictive tests for a wide range of disease predispositions [110,112,113,114,115]. There is little regulatory control of direct-to-consumer testing services under the In Vitro Medical Devices Directive [119], and during the COVID-19 pandemic several EU member states (including the UK [44]) prohibited their use and distribution due to concerns of the accuracy of these tests, especially as the sampling collection method (e.g., capillary blood collected by a finger-prick) used for some tests were designed to be performed by healthcare professionals (blood venous sample) and not unqualified individuals (e.g., the general public) [42,43,45,46,120]. Our identification and monitoring of direct-to-consumer testing services for SARS-CoV-2 assisted UK agencies by providing clear and valuable insight into the scale and range of tests and services available. Following a review by the Medicines and Healthcare products Regulatory Agency, providers of direct-to-consumer testing services are now able to offer this service in the UK, if the sample collection kit and test adhere to new guidance [42,46]. 

## 4. Discussion

### 4.1. Global Eruption of Diagnostic Technologies in the Wake of COVID-19

Here we present a state of the diagnostics landscape, based upon the data captured for MDx and IDx technologies, either approved or in the development phase for clinical use for SARS-CoV-2. In total 1608 diagnostic solutions (excluding direct-to-consumer service tests) were identified and tracked between 16 March and 16 October 2020. Our comprehensive dataset shows the tremendous speed and scale in which diagnostic innovations for SARS-CoV-2 were produced around the world. With the overall case-fatality rate escalating across the world, testing formed a key pillar of national strategies in response to the COVID-19 pandemic. Tracking the developments stage and regulatory status of diagnostic technologies emerging on the COVID-19 landscape was, therefore, critical in providing a deep insight and understanding of diagnostic solutions, especially at a time when healthcare systems and testing facilities were under immense strain and nations sought to rapidly expand their testing capacity. 

Our dataset highlights the highly variable rates of activities in the development of molecular-based and immunological-based diagnostics across different countries and on a continental scale. We know that countries who implemented large-scale testing [7,59,61] were heavily reliant on the deployment of developed technologies as the scale of the crisis became global and systemic [59,63,64,65,66]. Evidence appears to suggest that companies played a key role in enabling countries in Asia to rapidly roll-out large-scale testing programmes, by designing and manufacturing large volumes of diagnostics in these regions [59,62,63,64,65,66]. Our review of the literature coupled with the insights gained from our global dataset, appears to highlight that those countries with 1) limited IVD market infrastructure/research and development (R&D) capacity and 2) limited testing infrastructure (including access to diagnostic technologies, trained staff and testing facilities), were hampered in their strategic preparedness and implementation of diagnostic testing strategy’s [105,106,107,108,109]. 

RT-qPCR still remains the gold standard molecular-based technique employed by health providers for COVID-19 worldwide [2,9,20]. However, operational pressures in testing facilities at the start of the pandemic was a key driver for technological advancements in diagnostic solutions (including sampling techniques). These innovative solutions played a key role in various aspects of the diagnostic process including portable, faster and easy to use diagnostics to support efforts to control the spread of the infection. Some examples of diagnostic techniques include gene-editing technologies (e.g., CRISPR [121,122]), nanotechnology [123], smartphone-based tests [124] and wearable technologies [125]. The rapid spread of COVID-19 also caused a rise in the use of 3D-printing to address shortages in swabs [126,127,128] and other innovative sampling including the use of breath analysis (also known as breathomics [129]), in a rapid, non-invasive diagnostic solution to detect the virus [130,131]. Whilst these innovative technologies are considered key enablers, there is currently a lack of evidence that provides insights into the scale of their implementation and the outcomes from their adoption, nationally and internationally.

Changes to COVID-19 restrictions following the first wave of the pandemic led to a growing demand for rapid diagnostics from some national authorities, because of their ease of use, fast results and scalability [16,24,25,31,77]. The deployment of these tests was to enable the recovery of international travel, the reopening of educational institutions and workplaces, where mass testing became imperative to curb the rate of transmission. 

Our data does not provide insight on the efficacy (e.g., sensitivity and specificity) of diagnostics technologies; however, the validity of all SARS-CoV-2 diagnostic tests must be considered, especially as different nations have different standards in relation to the evaluation of tests [132]. Increased understanding of performance variability with existing diagnostics and standardization of performance test protocols will effectively increase accessibility to the most robust and accurate diagnostics solutions [133,134,135]. Further research, evaluation and a coordinated partnership among governments (including public health), regulatory agencies, industry, academia and clinicians across the world is critical in addressing these challenges both now and for future outbreaks [133,136]. 

### 4.2. Impact of SARS-CoV-2 Technological Innovation on Regulatory Approval

Regulatory agencies around the world responded rapidly to the evolving COVID-19 situation and ensuing challenges of regulating emerging diagnostic and other technological innovations (e.g., ventilators). We collected and verified the regulatory authorization data for more than 1000 diagnostic tests from regulatory agencies across the world including the United States, Canada, Singapore, Australia, Korea and Brazil [48,49,50,51,52,53,54,55,56]. In total, 529 approvals were authorized for MDx technologies compared to 521 for IDx technologies. Whilst Europe was behind Asia and North America in the scale of diagnostic solutions developed, this region recorded the second highest number of approvals issued. The enormity of the current climate caused members states in Europe, like many regions, to adapt their guidelines for diagnostics to accelerate their availability on the market. However, it has been postulated that the European framework for IVDs is weak in relation to technologies which are considered ‘low risk’, because it allows developers to independently self-certify that their SARS-CoV-2 diagnostics comply with the regulatory requirements (i.e., self-award CE mark) [119,137,138]. The introduction of the IVD Regulation (IVDR) which will replace the current Directives (IVDD) will mean that most ‘low risk’ IVDs will be re-classified into higher risk classes, therefore requiring the involvement of a designated organization (called a Notified Body), instead of self-assessments [119,137,138]. A major challenge for low- and middle-income countries evaluating SARS-CoV-2 diagnostics, is the lack of resources to deliver a robust regulatory system. As a result, many relied on the regulatory approvals from highly regulated countries and WHO’s Emergency Use Listing (EUL) [107,108]. Furthermore, their fragmented healthcare and regulatory systems weaken the incentive of companies to invest (e.g., develop and commercialize products) in such a small market. Despite African nations continuing to rely on internationally manufactured tests, they have collaborated to expedite the regulatory review and distribution of diagnostics [107,139,140] and increase testing efforts through global partnerships (e.g., knowledge exchange and training) [107,141,142]. 

## 5. Conclusions

The mass production of SARS-CoV-2 diagnostics has helped overcome bottlenecks associated with PCR-based detection and enhanced testing capacities during the pandemic. Nonetheless, the development of these tests has been complex and associated with challenges in relation to clinical accuracy, regulatory assessment, availability and implementation across differing healthcare environments around the world. Only time will tell how the IVD industry, healthcare services, governments and regulatory agencies around the world will further adapt to address evolving needs and the long-term impact of the pandemic. Importantly, the sharing of key learnings and best practices from the pandemic (including methods for the evaluations of diagnostics, development programmes and testing strategies) from across the world, will be critical in helping to build resilience across healthcare and regulatory systems against future health challenges. 

## Data Availability

Publicly available datasets were analyzed in this study. This data can be found here: http://www.io.nihr.ac.uk/report/covid-19-diagnostics/.
